# Supplementary material for: Comprehensive evaluation and characterisation of short read general-purpose structural variant calling software
Source: Nat Commun. 2019 Jul 19;10:3240. doi: 10.1038/s41467-019-11146-4 (PMC6642177; doi:10.1038/s41467-019-11146-4)
Supplement: Supplementary file 1 — Supplementary Information [file 41467_2019_11146_MOESM1_ESM.pdf]

Comprehensive evaluation and characterisation of short read general purpose structural variant calling software

Authors: Daniel L Cameron, Leon Di Stefano, Anthony T Papenfuss

Supplementary Figures

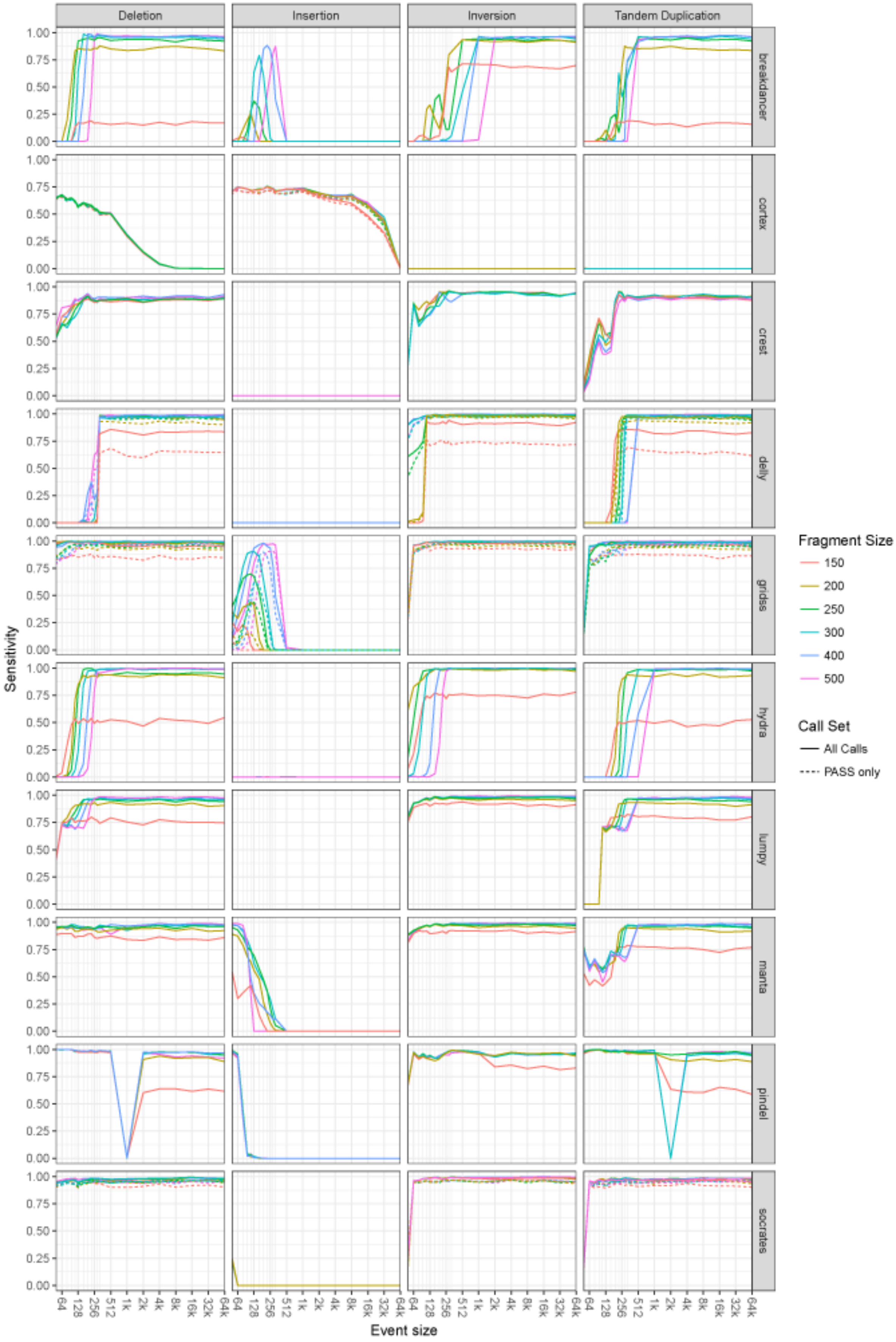
Deletion
Insertion
Inversion
Tandem Duplication
breakdancer
cortex
crest
delly
gridss
hydra
lumpy
manta
pindel
socrates
Sensitivity
1.00
0.75
0.50
0.25
0.00
Event size
64
128
256
512
1k
2k
4k
8k
16k
32k
64k
Fragment Size
150
200
250
300
400
500
Call Set
All Calls
PASS only

Supplementary Figure 1: Sensitivity of structural variant callers on simulated data across different event types for typical resequencing parameters (2x100bp, 60x depth) and varying fragment size.

High confidence "PASS" calls (dashed line) and all calls (solid lines) are shown where available. Simulation results represent an upper bound on caller performance on human sequencing data.

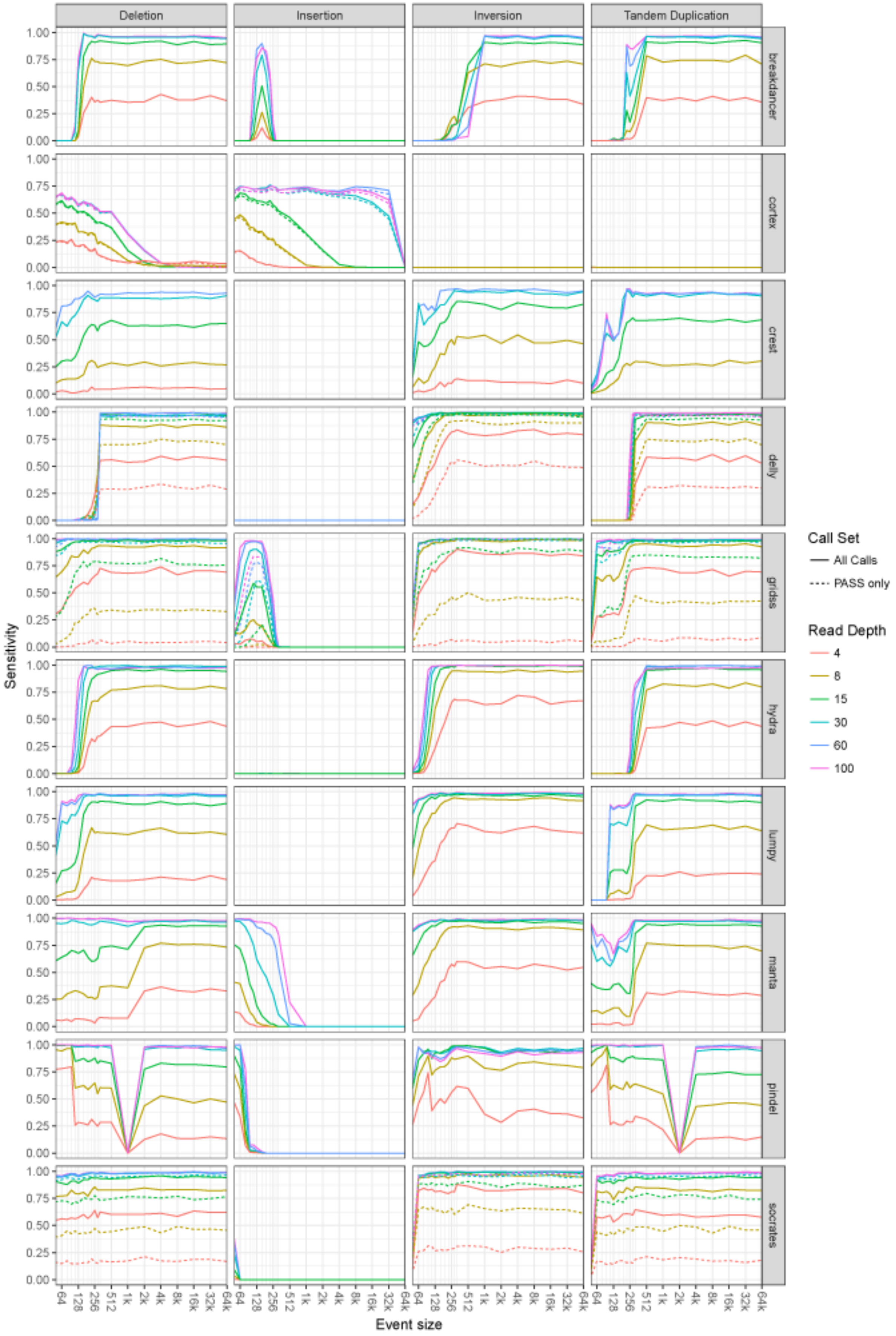
Deletion
Insertion
Inversion
Tandem Duplication
breakdancer
cortex
crest
delly
gridss
hydra
lumpy
manta
pindel
socrates
Sensitivity
Event size
0.00
0.25
0.50
0.75
1.00
64
128
256
512
1k
2k
4k
8k
16k
32k
64k
Call Set
All Calls
PASS only
Read Depth
4
8
15
30
60
100

Supplementary Figure 2: Sensitivity of structural variant callers on simulated data across different event types for typical resequencing parameters (2x100bp, 300bp fragment size) and varying sequencing depth.

High confidence "PASS" calls (dashed line) and all calls (solid lines) are shown where available. Simulation results represent an upper bound on caller performance on human sequencing data.

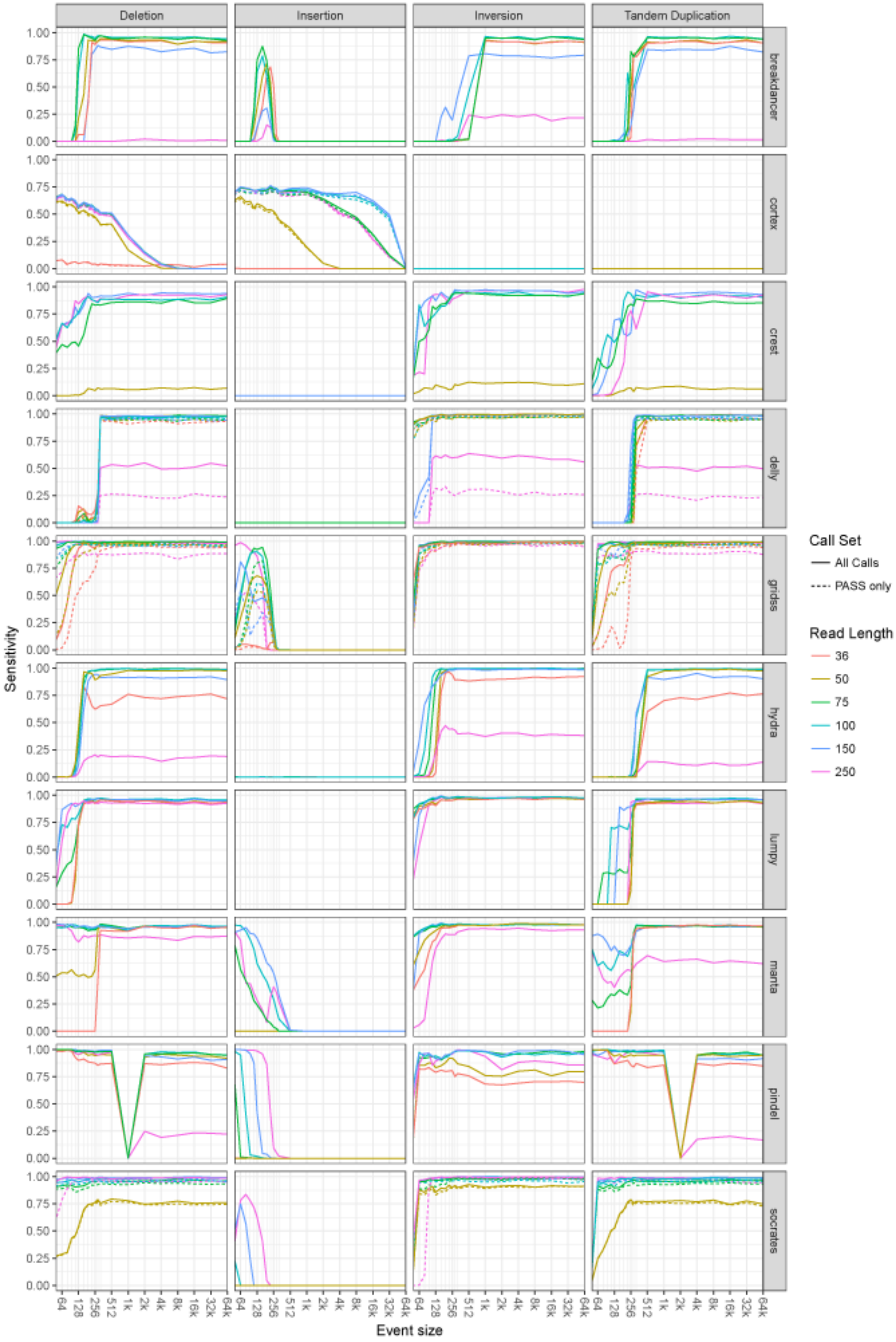

Deletion
Insertion
Inversion
Tandem Duplication
breakdancer
cortex
crest
delly
gridss
hydra
lumpy
manta
pindel
socrates
Sensitivity
Event size
Call Set
All Calls
PASS only
Read Length
36
50
75
100
150
250

Supplementary Figure 3: Sensitivity of structural variant callers on simulated data across different event types for typical resequencing parameters (60x depth, 300bp fragment size) and varying read length.

High confidence “PASS” calls (dashed line) and all calls (solid lines) are shown where available. Simulation results represent an upper bound on caller performance on human sequencing data.

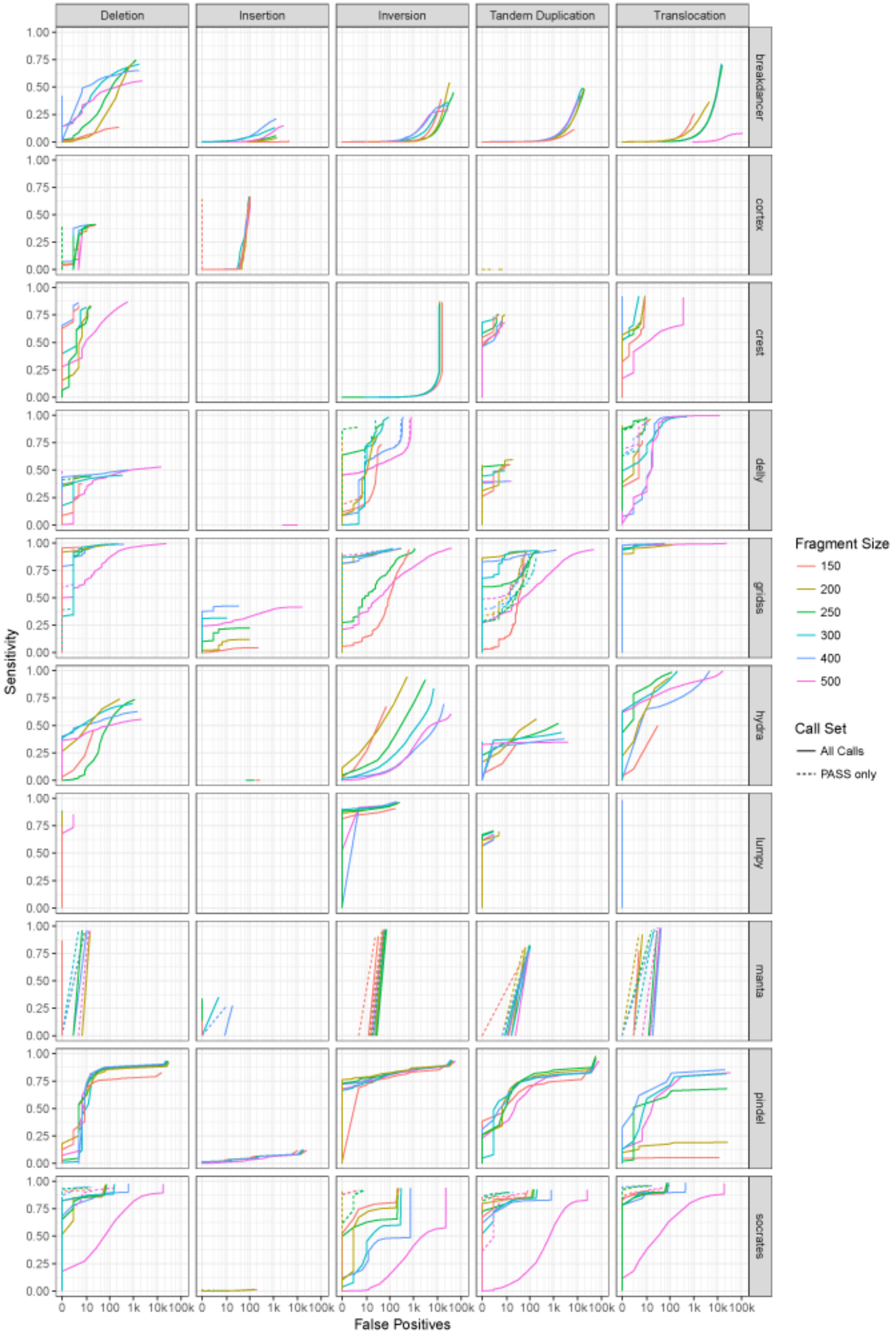
Deletion
Insertion
Inversion
Tandem Duplication
Translocation
breakdancer
cortex
crest
delly
gridss
hydra
lumpy
manta
pindel
socrates
Sensitivity
False Positives
0
10
100
1k
10k
100k
0.00
0.25
0.50
0.75
1.00
Fragment Size
150
200
250
300
400
500
Call Set
All Calls
PASS only

Supplementary Figure 4: Overall sensitivity vs false positives on simulated data across different event types for typical resequencing parameters (2x100bp, 60x depth) and varying fragment size.

High confidence “PASS” calls (dashed line) and all calls (solid lines) are shown where available. Translocation events were simulated through random rearrangement.

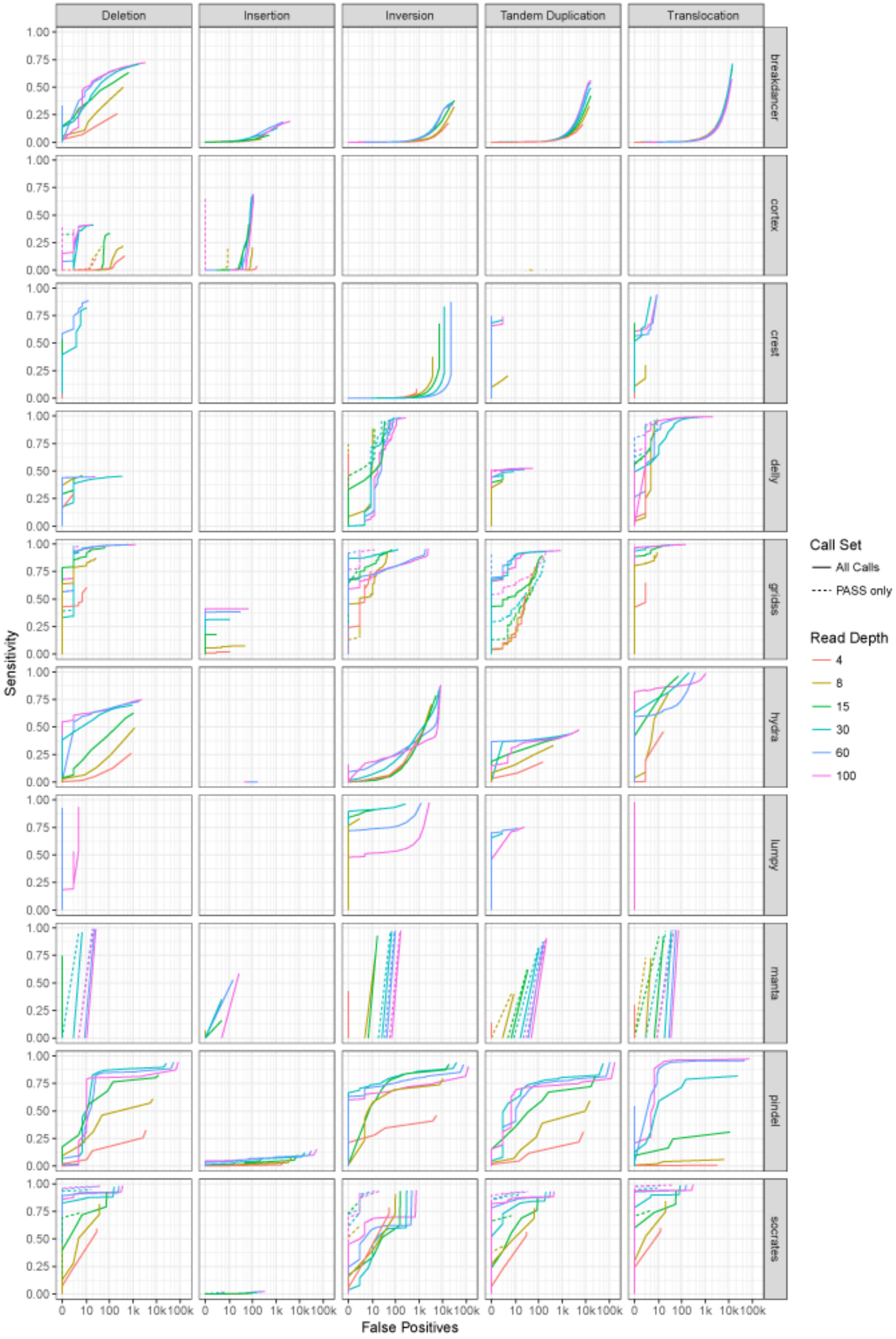

Deletion
Insertion
Inversion
Tandem Duplication
Translocation
breakdancer
cortex
crest
delly
gridss
hydra
lumpy
manta
pindel
socrates
Sensitivity
False Positives
0
10
100
1k
10k
100k
0.00
0.25
0.50
0.75
1.00
Call Set
All Calls
PASS only
Read Depth
4
8
15
30
60
100

Supplementary Figure 5: Overall sensitivity vs false positives on simulated data across different event types for typical resequencing parameters (2x100bp, 300bp fragment size) and varying sequencing depth.

High confidence "PASS" calls (dashed line) and all calls (solid lines) are shown where available. Translocation events were simulated through random rearrangement.

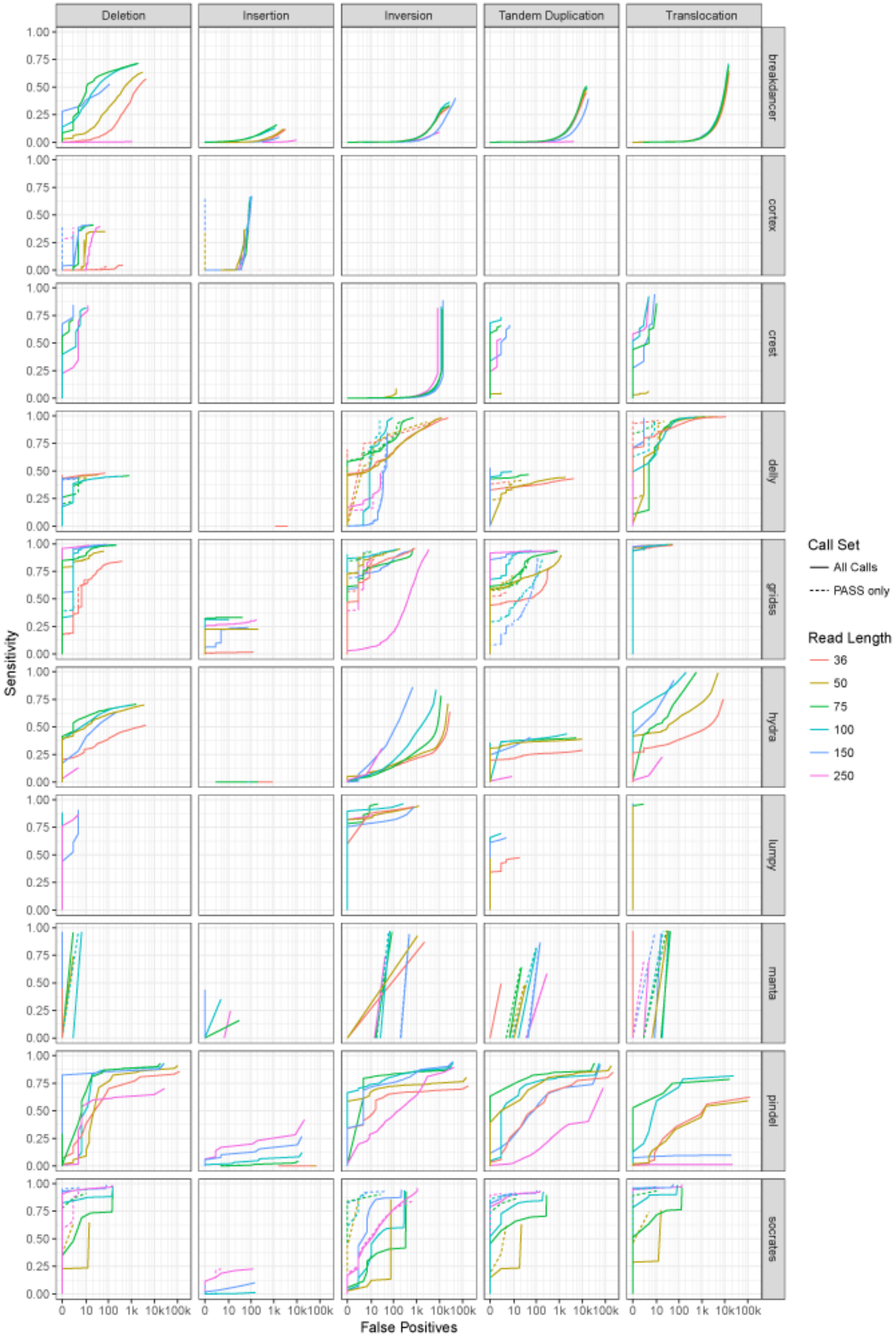

Deletion
Insertion
Inversion
Tandem Duplication
Translocation
breakdancer
cortex
crest
delly
gridss
hydra
lumpy
manta
pindel
socrates
Sensitivity
False Positives
0
10
100
1k
10k
100k
0.00
0.25
0.50
0.75
1.00
Call Set
All Calls
PASS only
Read Length
36
50
75
100
150
250

Supplementary Figure 6: Overall sensitivity vs false positives on simulated data across different event types for typical resequencing parameters (60x depth, 300bp fragment size) and varying read length.

High confidence “PASS” calls (dashed line) and all calls (solid lines) are shown where available. Translocation events were simulated through random rearrangement.

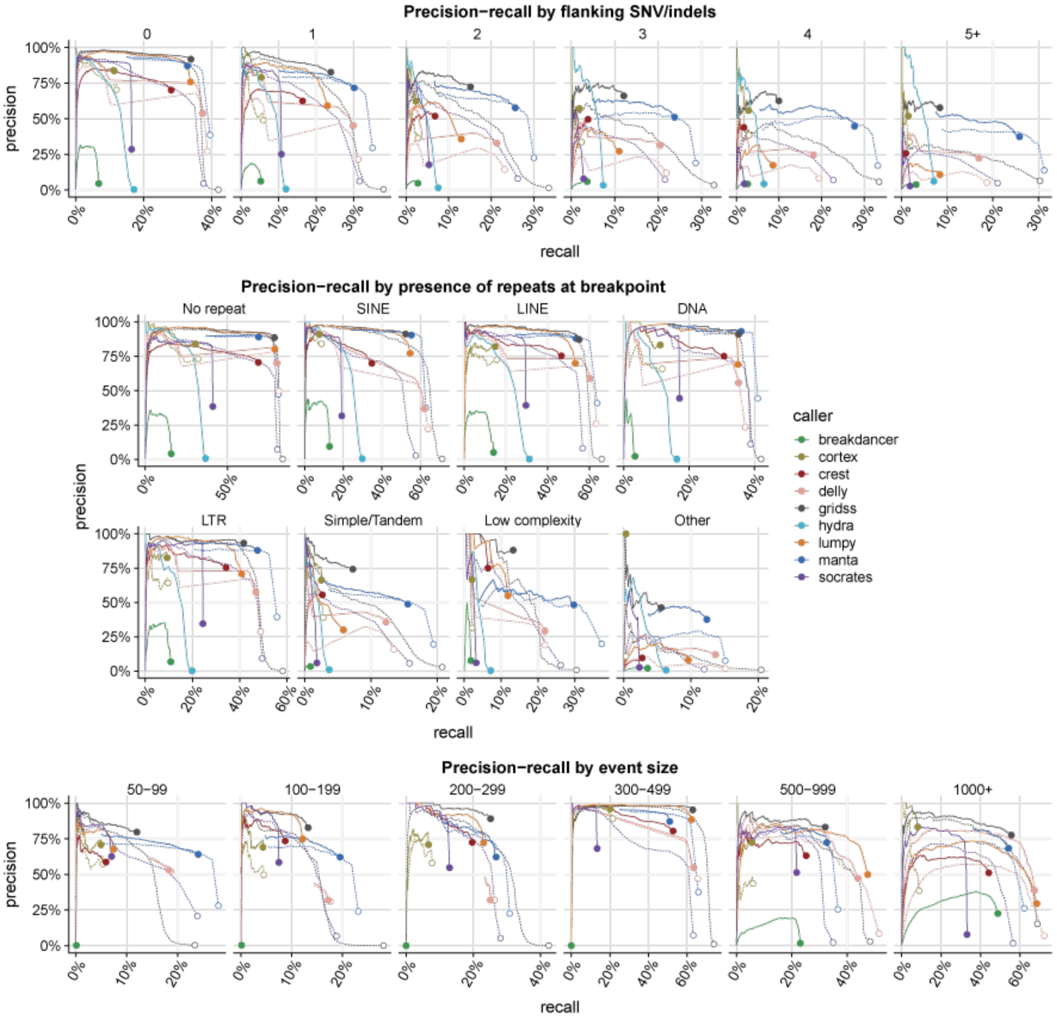

Supplementary Figure 7: Caller performance by sequencing context on the CHM1 cell line.

Caller performance trends are consistent with the NA12878 cell line results.

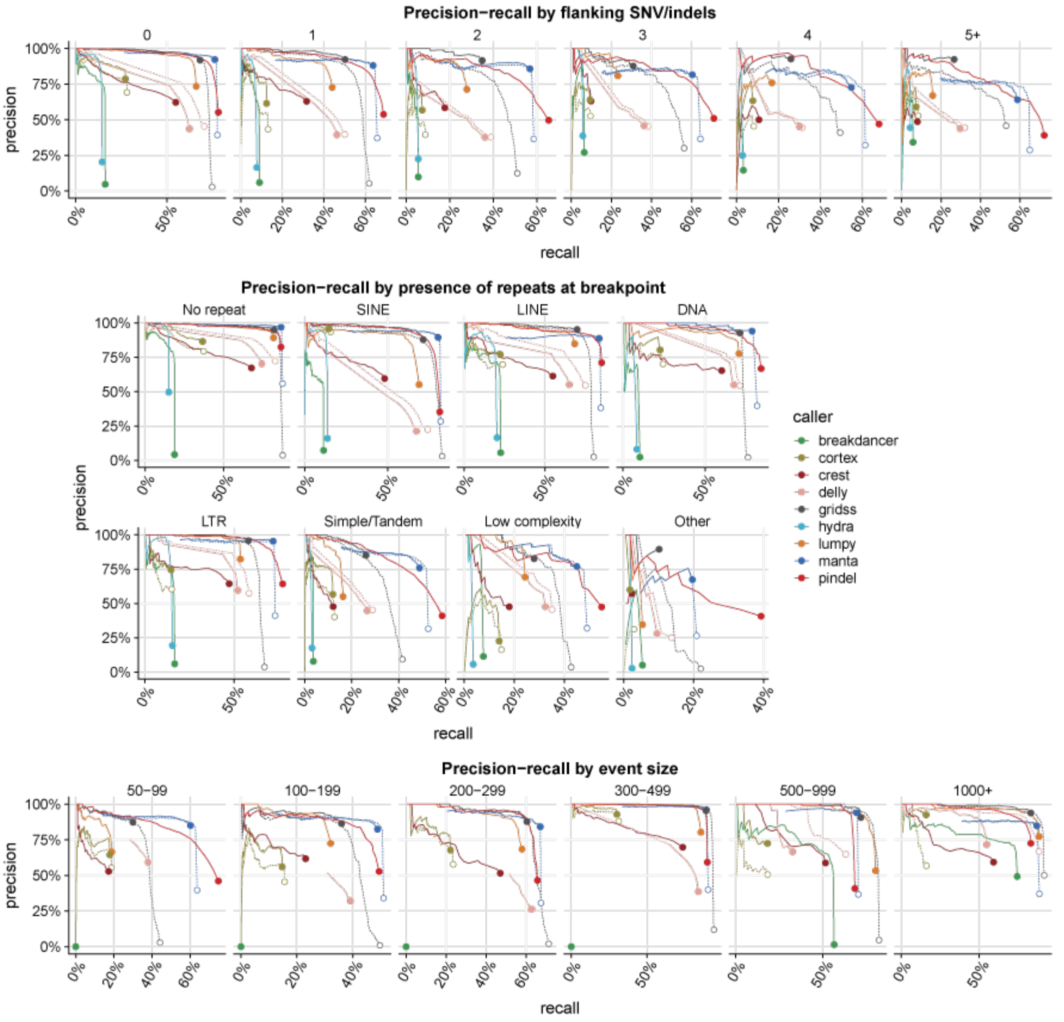

Supplementary Figure 8: Caller performance by sequencing context on the CHM13 cell line.

Caller performance trends are consistent with the NA12878 cell line results.

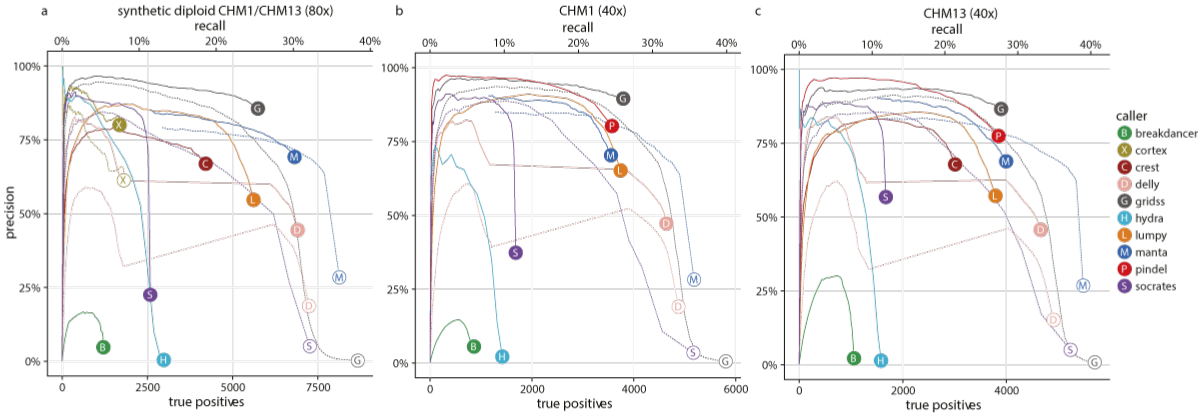

Supplementary Figure 9: Caller performance on synthetic CHM1/CHM13, CHM1 and CHM13 cell lines.

Unlike SNV detection, SV detection capability is independent of variant zygosity and is driven by variant haplotype coverage. Caller agreements trends are consistent with the NA12878 cell line results. In contrast to the NA12878, the CHM1 and CHM13 truth sets are comprehensive call sets. Around half of all structural variants are not detected by any caller. These missed calls are overwhelmingly in low complexity regions and simple repeats.

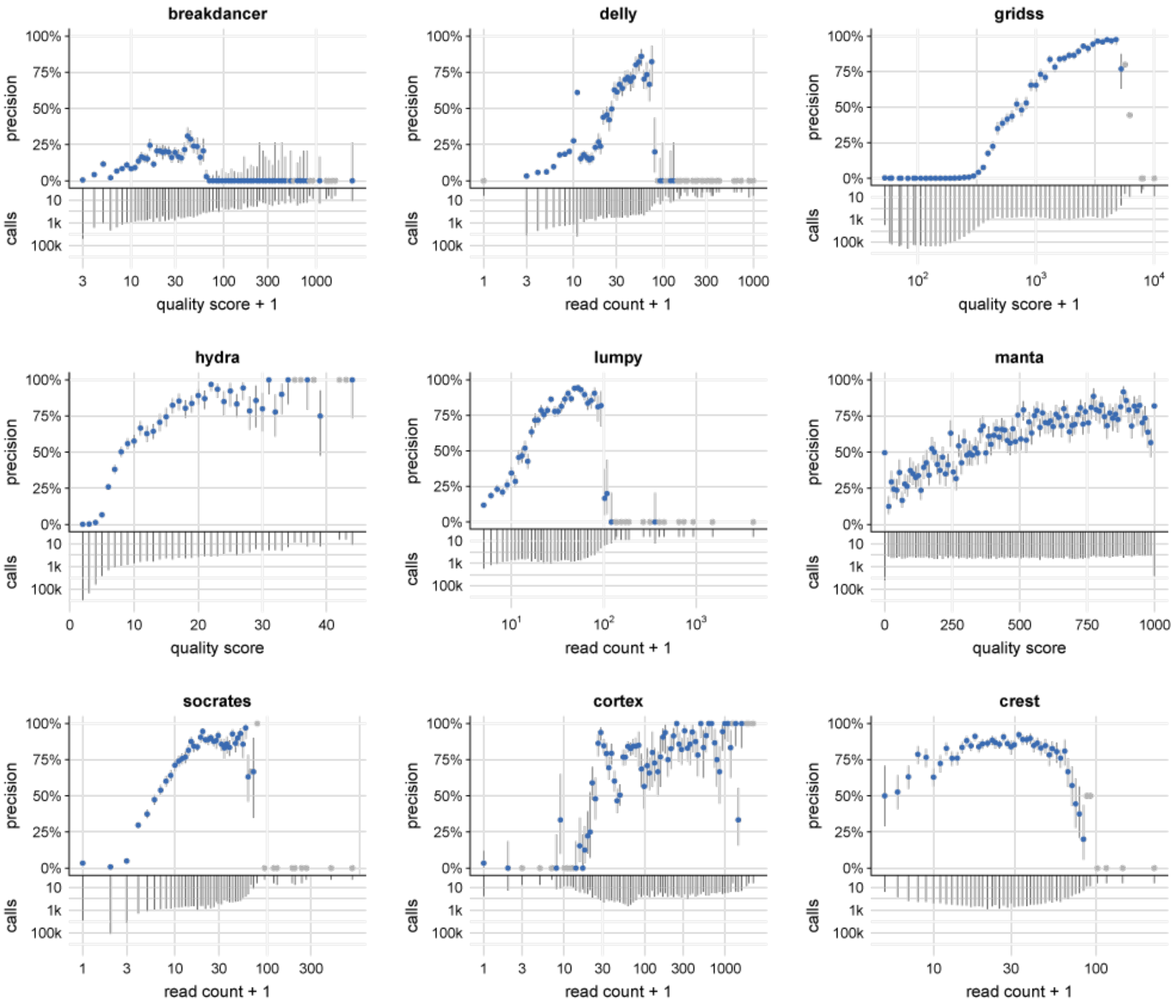

Supplementary Figure 10: Stratification of calls by supporting reads and variant quality score on the CHM1 cell line.

For each caller, the results for the CHM1 dataset were separated into 100 bins by either (log) read count or quality score, as indicated. For each bin, the precision (upper plot) and number

of calls falling within the bin (lower plot) was calculated. Grey bars indicate 95% binomial confidence intervals for the precision. Bins with 10 or fewer calls are colored grey and confidence interval bar omitted.

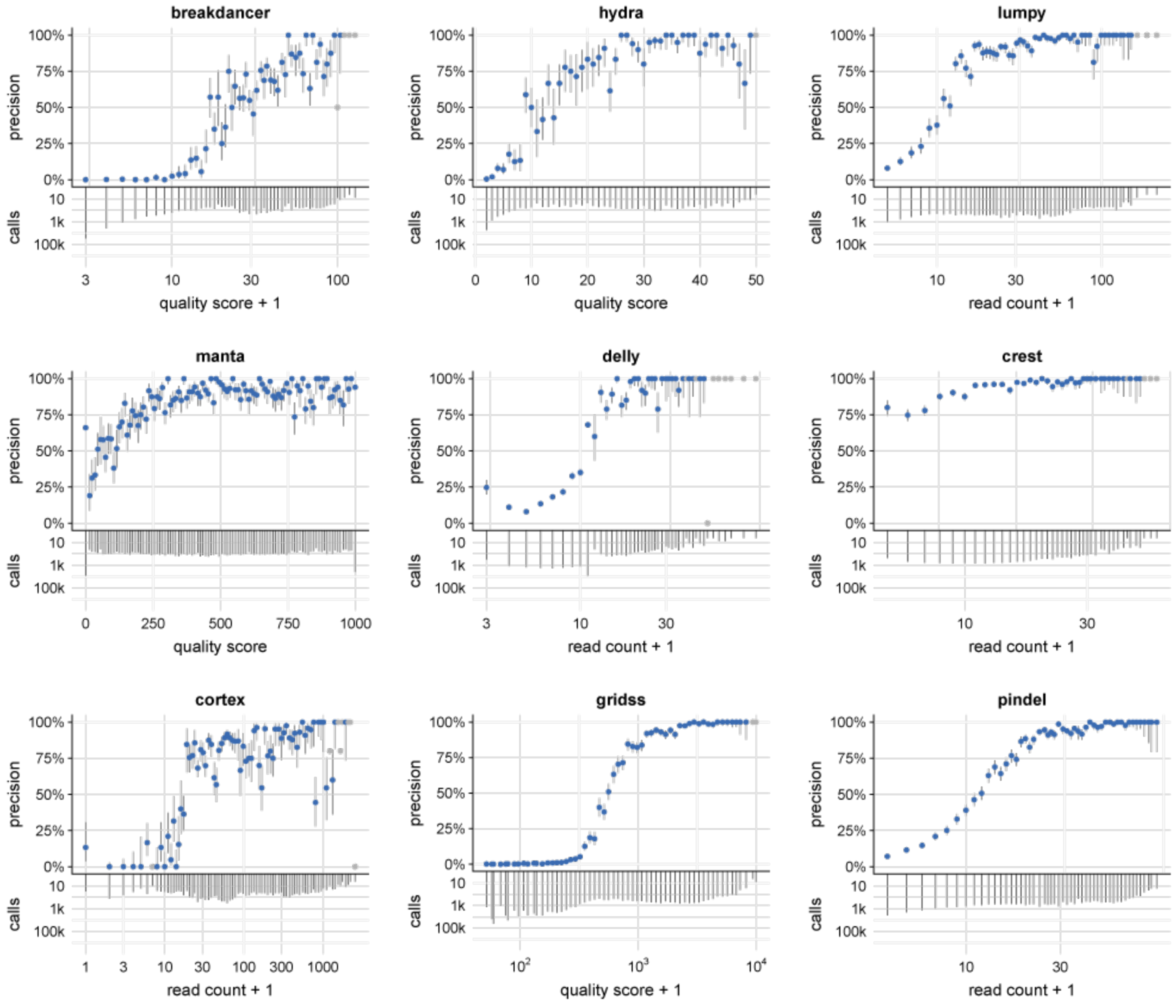

Supplementary Figure 11: Stratification of calls by supporting reads and variant quality score on the CHM13 cell line.

For each caller, the results for the CHM13 dataset were separated into 100 bins by either (log) read count or quality score, as indicated. For each bin, the precision (upper plot) and number of calls falling within the bin (lower plot) was calculated. Grey bars indicate 95% binomial confidence intervals for the precision. Bins with 10 or fewer calls are colored grey and confidence interval bar omitted.

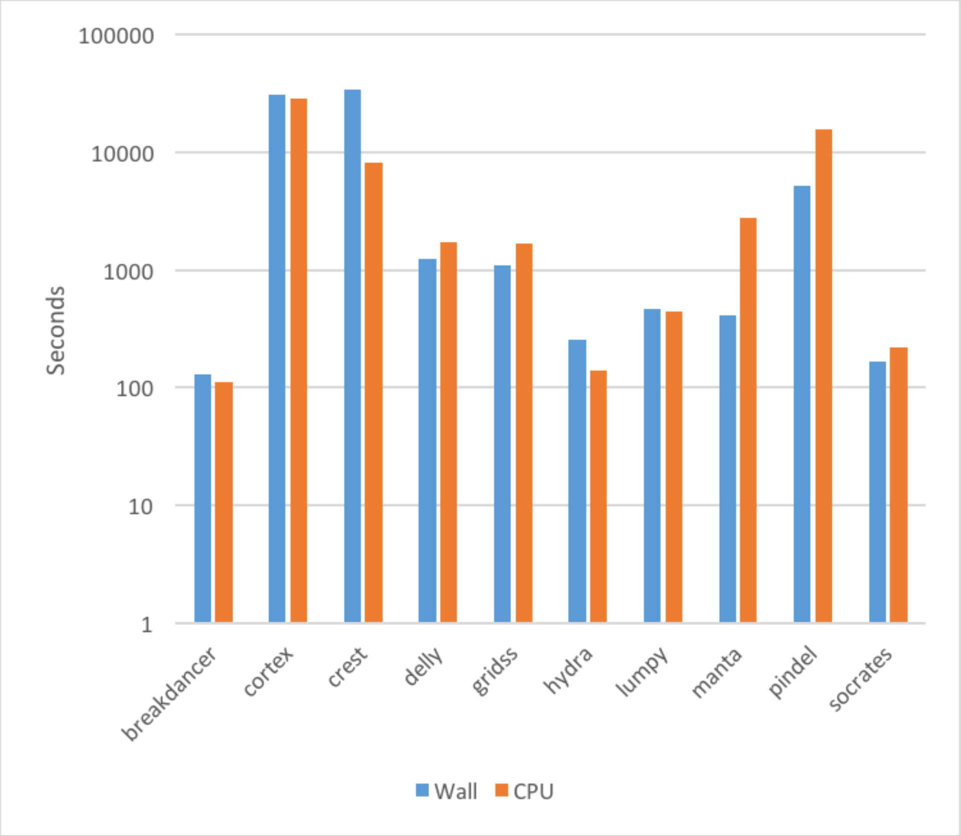

Supplementary Figure 12: Relative runtime performance of callers.

Runtime performance was measured using the Unix *time* command on a dual socket Xeon E5-2690v4 with 512GB of memory. Wall time indicates total elapsed seconds. CPU time indicates total CPU utilization and can be less than wall time for multi-threaded callers.

Supplementary Tables

Software	Year	First author	Input format	Output format	DP	OEA	SC	Read Depth	Assembly	Type	Multi-mapping	Description
VariationHunter	2009	Hormozdiari	DIVET	Custom	Y						Y	Chooses mapping locations of multi-mapping reads such that the number of SV calls is minimised.
GASV	2009	Sindi	BAM	Custom	Y							Computes all mutually consistent DP subsets (maximal cliques) based on fragment size threshold
Pindel	2009	Ye	BAM	VCF		Y	Y					Pattern growth algorithm finds split read mapping nearby anchoring mate read.<br>Event size limited by search window size.
Breakdancer	2009	Chen	BAM	Custom	Y							BreakDancerMax identifies regions containing more DPs than empirically expected.<br>BreakDancerMini calls small indels from read pairs BreakDancerMax considered concordant
HYDRA	2010	Quinlan	BAM	Custom	Y						Y	Greedy DP clustering.
VariationHunter-CR	2010	Hormozdiari	DIVET	Custom	Y							Improves VariationHunter by assuming diploid genome<br>Adds specialised transposon handling by aligning to RepBase consensus sequences

SVDetect	2010	Zeitouni	BAM	BED	Y							Uses sliding window to perform DP clustering
SVMerge	2010	Wong	BAM	Custom					Y	targeted		Targeted assembly validation of calls from BreakDancer, Pindel, SE Cluster, RDXplorer, and RetroSeq
SOAPsv	2011	Li	FASTQ	Custom					Y	de novo		Reference alignment of whole genome de novo assembly contigs. Homozygous variants < 50kbp only.
SRiC	2011	Zhang	FASTQ	Custom		Y						Split read identification through BLAT gapped alignments<br>Support threshold varies with indel size to control FDR
CREST	2011	Wang	BAM	Custom			Y		Y	targeted		Targeted CAP3 breakend assembly of SC clusters; BLAT alignment of assembled contigs
SVseq	2011	Zhang	SAM	Custom	Y	Y						Split read caller. FDR reduced by requiring DP support. Event size limited by search window size.
CommonLAW	2011	Hormozdiari	DIVET	Custom	Y						Y	Multi-sample extension of VariationHunter
ClipCrop	2011	Suzuki	BAM	BED			Y					Split reads identified by bwa alignment of SC bases
GASVPro	2012	Sindi	BAM	Custom	Y			Y			Y	Combines GASV calls with read depth signal.<br>MCMC placement of multi-mapping reads
SVseq2	2012	Zhang	BAM	Custom	Y		Y					Split read mapping of soft clipped reads to only regions with DP support
SVM2	2012	Chiara	Unknown	Unknown	Y							Support vector machine used to call DP events. SVM trained by on simulated indels.<br>Software no longer available

PRISM	2012	Jiang	BAM	Custom	Y	Y						Split read mapping to regions with DP support, or small indels
DELLY	2012	Rausch	BAM	Custom	Y	Y						Breakpoint position of DP calls refined by searching for supporting split reads.
CLEVER	2012	Marschall	BAM	VCF	Y						Y	Clustering of all read pairs to identify discordant clusters
SVMiner	2012	Hayes	BAM	Custom	Y							DP clustering with event type uncertainty
cortex_var	2012	Iqbal	FASTQ	VCF					Y	de novo		De novo assembly, LASTZ mapping of contigs
BreakPointer	2013	Drier	Custom	Custom	Y	Y						Breakpoint position of DP calls refined by searching for supporting split reads.<br>Requires BAM & dRanger rearrangment predictions as input
SV-M	2013	Grimm	BAM	Custom		Y						SVM trained sanger validation. Heterozygous events only. Event size limited to < 5kbp.
PeSV-Fisher	2013	Escaramís	BAM	Custom	Y			Y				Greedy DP clustering. RD used for variant classification and annotation
Bellerophon	2013	Hayes	BAM	Custom	Y		Y					Soft clipped reads refine DP clusters.<br>Interchromosomal only.
Meerkat	2013	Yang	BAM	VCF	Y	Y	Y				Y	Greedy DP clustering. BWA/BLAST split read identification.
SoftSearch	2013	Hart	BAM	VCF	Y		Y					Combined cluster of SR and DP.
Socrates	2014	Schröder	BAM	Custom			Y			targeted		Split reads identified by bowtie2 alignment of SC cluster consensus sequences
breseq	2014	Barrick	BAM	HTML			Y	Y				Split reads identified from bowtie2 multi-mapping reads.<br>Haploid microbial genomes

LUMPY-sv	2014	Layer	BAM	VCF	Y		Y	Y				Signals converted to probability intervals then combined
SVFinder	2014	Yang	BAM	BED	Y							Greedy DP clustering
Gustaf	2014	Trappe	FASTQ	VCF			Y					Multi-split read alignment
TIGRA-ext	2014	Chen	BAM	VCF					Y			Targeted assembly validation of calls from BreakDancer, Pindel, or DELLY
laSV	2015	Zhuang	FASTQ	VCF					Y	de novo		De novo assembly, bwa mapping of contigs<br>Validational bwa alignment of reads to reference + putative SVs
AsmVar	2015	Liu	MAF	VCF					Y	de novo		Assembly-versus-assembly alignment of reference and de novo assemblies
RAPTR-SV	2015	Bickhart	BAM	Custom	Y	Y	Y				Y	Uses VariantionHunter clustering and multi-mapping resolution.<br>SR identification using MrsFAST alignment of 50/50 split of read bases.
MetaSV	2015	Mohiyuddin	BAM	VCF					Y	targeted		Targeted assembly of calls from BreakDancer, Pindel, CNVnator, BreakSeq, and soft clips
BreaKmer	2015	Abo	BAM	Custom	Y	Y	Y		Y	targeted		Targeted assembly of misaligned reads. Not suitable for whole-genome
SoftSV	2016	Bartenhagen	BAM	Custom	Y		Y		Y	targeted		Targeted OLC assembly of SC reads. Target regions identified by DP clusters
Hydra-Multi	2015	Lindberg	BAM	Custom	Y						Y	Multi-sample extension of HYDRA
Wham	2015	Kronenberg	BAM	VCF	Y		Y					Clustering of SC, DP, and bwa split read alignments. Targeted validating breakpoint assembly.

SV-Bay	2016	Iakovishina	BAM	Custom	Y			Y				Bayesian model using read depth, mappability, and GC bias to filter candidate DP clusters.
GRIDSS	2017	Cameron	BAM	VCF	Y	Y	Y		Y	alignment guided	Y	Whole genome breakend assembly. Clustering of contigs, SR, DP.
Manta	2016	Chen	BAM	VCF	Y		Y		Y	targeted		Clustering of SC, DP. Targeted validating breakpoint assembly.
Sprites	2016	Zhang	BAM	Custom								Split read mapping of whole soft clipped reads to regions with DP support

Supplementary Table 1: General purpose SV callers published since 2009. Benchmarked callers are highlighted in green. Callers for which full results could not be obtained are highlighted in yellow.

Supplementary Notes

Excluded callers

A number of structural variant callers were excluded from analysis for the following reasons:

CLEVER: On simulated data, CLEVER called many fewer results than expected (41 deletion calls, 0 for other events). Through personal correspondence with the software author it was determined that all published versions (1.1, 2.0rc1 and 2.0rc3) contained critical bugs causing either program failure, or call failure). As the recommended solution to compare against the most recent unstable, unreleased development version lacking both version and release information is not appropriate for reproducible evaluation, CLEVER results were excluded.

VariationHunter: No results for VariationHunter could be obtained. VariationHunter crashed with a "Segmentation fault (core dumped)" error on all simulations. VariationHunter could not be run on NA12878 in a timely manner due to requirement of in excess of 20,000 hours of computation time required to run mrfast on the 50x whole genome sequencing data set.

SOAPsv: A script converting the user guide (10 pages of instructions containing 76 different steps) was not able to generated in the two days allotted. The number of steps and the requirements to recompile a new version of the software for every sample due to the presence of hard-coded file path in the source code indicates that SOAPsv was not designed for general purpose usage.

GASVPro: Results were significantly worse than expected for a read pair based caller. Upon investigation it was found that the LLR scoring used by GASV excessively favoured very large events when using a single input BAM. Combined with a filter removing overlapping events, most true positive GASV calls were filtered by the cluster pruning algorithm. In NA12878, a

false positive deletion call from chr1:32,060,879 to chr1:243,114,737 with a LLR of 1.4×10^{10} resulted in the removal of all chr1 deletion calls within this interval, thus removing most chr1 calls. Under these circumstances, it was decided that GASVPro results were not representative of read pair-based caller performance and results were excluded.

Caller-specific behaviour on simulated typical resequencing data

To determine the performance of the variant callers for parameters typical of most current resequencing projects, we first examined a slice through our multi-dimensional simulation dataset corresponding to 2x100bp read sequencing, a fragment size distribution 300bp±10% (mean 300bp; standard deviation of 10%), and 30x coverage. We then evaluated the sensitivity for each event size (Supplementary Figure 2), as well as generating overall Receiver Operating Characteristic-like (ROC-like) curves for each event type (Supplementary Figure 5). These were generated by ordering predictions using the caller-reported variant quality score or, if no quality score was reported, using the caller-reported total number of reads supporting the variant.

BreakDancer: Although the sensitivity of BreakDancer loosely matches that expected of a discordant read pair based caller, BreakDancer exhibits a high false discovery rate across all event types. For inversions and tandem duplications, the event size detection range is smaller than that of deletions. Reduced inversion performance could be explained by the presence of two underlying breakpoints, but with tandem duplications consisting of only a single breakpoint, and other PE callers not exhibiting this behaviour for either event type, this reduced detection capability cannot be explained on theoretical grounds.

Cortex: As a de novo assembly based caller, cortex should in theory be capable of detecting all events across all event sizes. Unfortunately, the results from cortex do not live up to the theoretical expectations. Across all events, the maximum sensitivity of cortex does not exceed 75%, the lowest of any of the callers evaluated. While the default maximum events of 64,000bp explains the dip in performance on large insertions and the lack of breakpoint detection capability, it does not explain the gradual reduction in deletion sensitivity, the extremely low sensitivity for insertion events, nor the lack of large tandem duplication calls. Whilst specificity is low for inversions and tandem duplications, cortex specificity on insertion and deletion events is good.

CREST: Similar to cortex, CREST does not reach 100% sensitivity, instead asymptoting at around 90% sensitivity. CREST does not detect insertions so no insertion calls are made and specificity is good except for inversions where false positive calls are made before true positives.

DELLY: Event size range detection is limited by the PE breakpoint identification stage. Insertions are not called. Sensitivity and specificity are good for events that can be detected. Curiously, DELLY can detect smaller inversions than it can deletions or tandem duplications.

GRIDSS: With the highest F-score for breakpoints and deletions, inversions, and tandem duplications larger than 50bp, GRIDSS performs well. The trough in insertion sensitivity around 64bp matches the cross-over point between SR and PE detection capability, as can be seen from the SOCRATES and BreakDancer insertion sensitivities. Overall ROC-like performance on inversions and tandem duplications is reduced due to a significantly higher FDR for small events (<50bp) than for large events.

HYDRA: HYDRA performance is similar to that of DELLY, albeit with lower specificity. Insertion and tandem duplication event size detection is worse than DELLY but the size of the minimum deletion detectable by HYDRA is less than half that of DELLY.

LUMPY: Although LUMPY's minimum detectable event size is the worst of SR callers, it still outperforms PE-only callers and does so at a very low FDR. Notably, LUMPY made no false positive calls on either the deletion or breakpoint call sets. As with CREST, DELLY, and HYDRA, LUMPY does not support insertion calls.

manta: As a caller incorporating SR, PE, and assembly, manta performance is similar to that of GRIDSS even though their assembly approaches are quite different. Manta exhibits a smoother loss of sensitivity with insertion size as well as a longer maximum detectable insertion size. Unfortunately, this appears to come at the cost lower specificity across all event types, with the dip in tandem duplication sensitivity around 64bp considerably more pronounced in manta than in GRIDSS.

Pindel: Excluding large novel insertions, Pindel has the largest event size detection range of any of the callers evaluated and is the only caller that can reliably call events all the way down to 1bp in size. This sensitivity comes at the cost of a moderately high FDR and Pindel does not perform well when detecting arbitrary breakpoints. Curiously, Pindel exhibits a complete loss of sensitivity for 1kbp deletions as well as 2kbp tandem duplications.

SOCRATES: As a soft clipped based SR caller, the sensitivity of SOCRATES matches the theoretical expectations of a SC SR caller. As with GRIDSS, SOCRATES suffers from a high FDR for small inversion, and performs well on arbitrary breakpoint detection.

samtools: Results for samtools/bcftools have been included as representative of the detection capability of a typical SNV/small indel caller. Notably, the deletion detection range of samtools overlaps with that of SR and assembly based callers but not with callers primarily relying on PE support. A similar gap exists with insertions, although large insertions would still rely on de novo assembly or a specialised caller. Due to multiple alternative encodings of equivalent inversion and tandem duplication events in VCF, a straight-forward union of variant calls between samtools and any of the evaluated tools would not be sufficient and significant downstream data processing would be required regardless of the tools used.

Caller-specific read length behavior dependent on coverage

Of the PE-based callers, HYDRA most closely follows the expected behaviour of improved detection range with increased coverage. Unexpectedly, DELLY deletion and BreakDancer inversion display the opposite trend with higher coverage resulting in a more abrupt detection range cut-off threshold. For SR-based callers, the detectable event size range does not change with coverage, although there was a preferential drop in sensitivity toward the edge of the detectable size range. As expected, GRIDSS, LUMPY, and manta all display a drop in sensitivity at the PE cut-off point.

Pindel displays unusual behaviour. Firstly, a lack of 1kbp deletion and 2kbp tandem duplication calls is present at all levels of coverage. Secondly, the abrupt change in sensitivity at 100bp indicates that Pindel treats events smaller than the read length differently to those larger than the read length. In a similar way, the drop in the sensitivity of manta for medium size deletion

and tandem duplications only is not present for any other caller, nor is it readily explained by the published algorithm.

Caller-specific read length behavior dependent on fragment size

To examine the effect of read length on caller performance, we varied read length from 36 to 250bp. Different read lengths resulted in drastically different behavioural changes between callers (Supplementary Figures 3, 6). For PE callers, apart from changes in insertion sensitivity, performance was relatively unaffected by read length until the reads were sufficiently long that the majority of fragments contained overlapping reads. For 2x250bp sequencing of 300bp fragments, all PE based callers performed poorly with maximum sensitivity ranging from a bit over 50% for DELLY, to around 2% for BreakDancer.

Assembly-based callers exhibited severely degraded performance on reads 50bp or shorter. In the cases of cortex and CREST, where assembly is required for variant calling, this resulted in an almost complete loss of variant calls for 36bp reads. For GRIDSS and manta, this loss was restricted to certain event types and sizes as variant calls could be made from SR and/or PE support even in the absence of assembly.

Similar to assembly-based callers, SR callers also exhibited a drastic drop in sensitivity for reads 50bp or shorter. This drop can be explained by the reduction in maximum soft clip length. Since aligners require a minimum read length for alignment, soft clips below this length cannot be re-aligned. An as OEA SR caller, Pindel does not suffer this issue and is relatively unaffected by read length change, although again, Pindel failed to call 1kbp deletions and 2kbp tandem duplications under any conditions. Pindel large deletion and tandem duplication sensitivity above 1kbp was particularly poor for 2x250bp, suggesting that the Pindel algorithm

has evolved from the published OEA-only algorithm and now uses discordant read pairs to seed the search locations of nearby OEA. Such an algorithm would allow Pindel's missing calls to be plausibly explained by a bug in the event size algorithm choice logic of Pindel.

As expected, longer reads increase the maximum detectable event size of samtools and allows longer insertions to be detected for SR callers, assemblers are relatively unaffected by read length once assembly can be reliably performed, and the small window of detectable event sizes shifts with read length for PE callers.

When considering events larger than 50bp, GRIDSS retained the best mean sensitivity and highest mean F score for all event type except insertions for which cortex outperformed all over callers due to the underlying de novo assembly approach.

Caller-specific fragment size behavior dependent on fragment size

Next, to assess the effect of library fragment size distribution, the mean was varied from 150bp to 500bp with a standard deviation of 10%. Compared to changes in coverage or read length, the effect of fragment size was more isolated (Supplementary Figures 1, 4).

Just as observed with increasing read length, decreasing fragment size and the occurrence of overlapping read pairs significantly reduce PE caller performance. Although this was less pronounced at 2x100bp with 150bp fragments than at 2x250bp with 300bp fragments, the reduction in sensitivity was discernible across all PE callers. For the remaining non-overlapping fragment lengths, increasing the fragment size increased the minimum detectable event size as expected, except for deletions detected with DELLY, which retained the abrupt cut-off at 300bp.

As expected for variant callers that do not incorporate any read pairing information, samtools and cortex were unaffected by fragment size with the exception of a small reduction in large insertion sensitivity for cortex. In contrast, although theoretically they should produce identical results for all fragment sizes, both CREST and SOCRATES results shows signs of fragment size dependence, with both unexpectedly exhibiting minor variations in sensitivity and a significantly increased false positive rate for 500bp fragments.

Pindel again showed signs for PE dependence for events over 1kbp, and a lack of detection capability of 1kbp deletions and 2kbp tandem duplications, except this time, Pindel was capable of detecting 2kbp tandem duplication but only for fragment sizes less than 300bp.

Of the remaining callers, GRIDSS proved to be the most robust to fragment size changes with the drop in sensitivity significantly less for GRIDSS than for both manta and lumpy. Curiously, GRIDSS exhibited the same increase in false discovery rate displayed by CREST and SOCRATES, but only for low confidence events not supported by GRIDSS assembly. For insertions, only GRIDSS and BreakDancer showed the improvement in maximum detectable event size expected from increasing fragment size.

When considering events larger than 50bp, GRIDSS retained the best mean sensitivity and highest F mean score for all event types except cortex on insertions, and manta on inversions which, even with lower sensitivity, exceeded the GRIDSS F score.

Supplementary Discussion

Call matching logic

Unlike SNVs and small indels which can be left-aligned for an unambiguous representation, in general, SVs do not have a unique representation in VCF. An insertion, deletion or tandem duplication can be represented directly in the VCF ALT field using the variant sequence, using the symbolic INS/DEL/DUP ALT notation, or using the breakend notation introduced in VCF version 4.3. In the case of tandem duplications, the variant can be represented not only as a tandem duplication but can also be represented as an insertion of the duplicated sequence. Complicating this further are the inherit positional ambiguities introduced due to breakpoint sequence homologies as well as those introduced through imprecise calling of variant using supporting read pairs only. These positional ambiguities must be taken into account when matching variant calls and we have been generous in our matching criteria so as to not penalize read pair based callers. Unfortunately, as soon as imprecise call matching is performed, matched calls are no longer transitive. If calls A and B match, A and C match, it does not follow that B and C match. This complicates consensus calling considerably as such situations must be resolved when generating a consensus call set.

Additional complexity arises in repetitive regions. In such regions, expansion or contraction of repeat counts, such as a SINE tandem duplication expanding to 3 SINE repeats, can be reported as seemingly independent calls. An insertion at the start of the first SINE element and a tandem duplication of the second SINE element both result in the same sequence even though there is no overlap between the calls. Correctly matching such calls require full haplotype sequence reconstruction and comparison, a capability not present in any current SV call comparison tool. Such a tool would make a valuable contribution to the structural variant bioinformatics community.
