# Supplementary material for: Comprehensive evaluation and characterisation of short read general-purpose structural variant calling software
Source: Nat Commun. 2019 Jul 19;10:3240. doi: 10.1038/s41467-019-11146-4 (PMC6642177; doi:10.1038/s41467-019-11146-4)
Supplement: Supplementary file 4 — Description of Additional Supplementary Files [file 41467_2019_11146_MOESM4_ESM.pdf]

Description of Additional Supplementary Files

Supplementary Data 1:

Precision, FDR, sensitivity, and F-score for all x of y ensemble call sets for NA12878, CHM1, CHM13, CHM1/CHM13 synthetic diploid, and HG002.
